# The involvement of patients in developing clinical guidelines

**DOI:** 10.1186/1750-1172-7-S2-A13

**Published:** 2012-11-22

**Authors:** Kay Parkinson

**Affiliations:** 1 Alström Syndrome UK, 49 Southfield Avenue, Paignton, South Devon, TQ1 1LH, UK

## 

My two children were diagnosed with Alström Syndrome when aged 15 and 18 after a long battle to get their multiple medical conditions recognised as a syndrome. After diagnosis it was apparent that there were no experts in the condition in the UK and little information. To address this I founded the charity AS UK to bring together the 7 known cases with the doctors who had worked with my children. Their experience of the disease increased overnight primarily through talking with parents and patients who were the only experts in the disease. The first meetings were held in a hotel, later we moved to Torbay hospital using equipment at the weekend when no one else was using it. The ethos of listening to patients and parents as experts was fundamental to the development of the clinics. In 2006 we were successful in getting the NHS National Specialised Commissioning Group to recognise the importance of the clinics and they now fund children’s clinics at the Birmingham Children’s Hospital and adult’s clinics at the Queen Elizabeth Hospital Birmingham. AS UK was the first charity to be funded as an equal partner with the two hospitals. Today 55 patients are seen at the two clinics and expertise continues to grow. In 2009 AS UK was successful in gaining Big Lottery Medical and Scientific Funding and research has started. A database has been set up and tissue bank begun. In 2010 AS UK became a partner in an EU wide project with two other rare conditions EURO-WABB (Wolfram, Alström and Bardet Biedl) The overall aim is for this register to be a key instrument to increase knowledge on these rare diseases, improve the lives of affected people through better management, and to develop clinical research. In 2011 AS UK started an Asian mentoring scheme in response to the high number of Asian patients in families whose culture practice consanguinity. From the initial 3 Asian patients known to us we now know of 24 patients most are related. This is an area we are developing further. From small beginnings this patient led initiative has shown how an ultra-rare condition can go from obscurity to gaining NHS specialised clinical services, National and European research projects, medical handbooks, patient information, clinical guidelines and improved quality of life for patients.

